# Revealing Rotational Characteristics of the Uniflagellate Mutant of *Chlamydomonas reinhardtii* through DeepLabCut-Based Autotracking

**DOI:** 10.17912/micropub.biology.001535

**Published:** 2025-04-21

**Authors:** Azusa Kage, Ken H. Nagai, Takayuki Nishizaka, Kenta Ishimoto

**Affiliations:** 1 Graduate School of Engineering, Muroran Institute of Technology, Muroran, Hokkaido, Japan; 2 School of Materials Science, Japan Advanced Institute for Science and Technology, Nomi, Ishikawa, Japan; 3 Department of Physics, Gakushuin University, Toshima-ku, Tokyo, Japan; 4 Department of Mathematics, Kyoto University, Kyoto, Japan

## Abstract

Tracking eukaryotic flagella and cilia often requires manual clicking, even in the age of digital imaging. We developed an autotracking method using DeepLabCut, a CNN-based, marker-less tracking tool originally designed for animal behavior. Applying this method, we uncovered rotational characteristics of *Chlamydomonas reinhardtii* *uni1* , a uniflagellate mutant. Live *uni1* cells predominantly rotated counterclockwise under a coverslip when viewed from above, whereas demembranated models exhibited slower, more clockwise rotation. These differences likely stem from alterations in the three-dimensional flagellar waveform.

**Figure 1. Rotational Characteristics of uni1:**
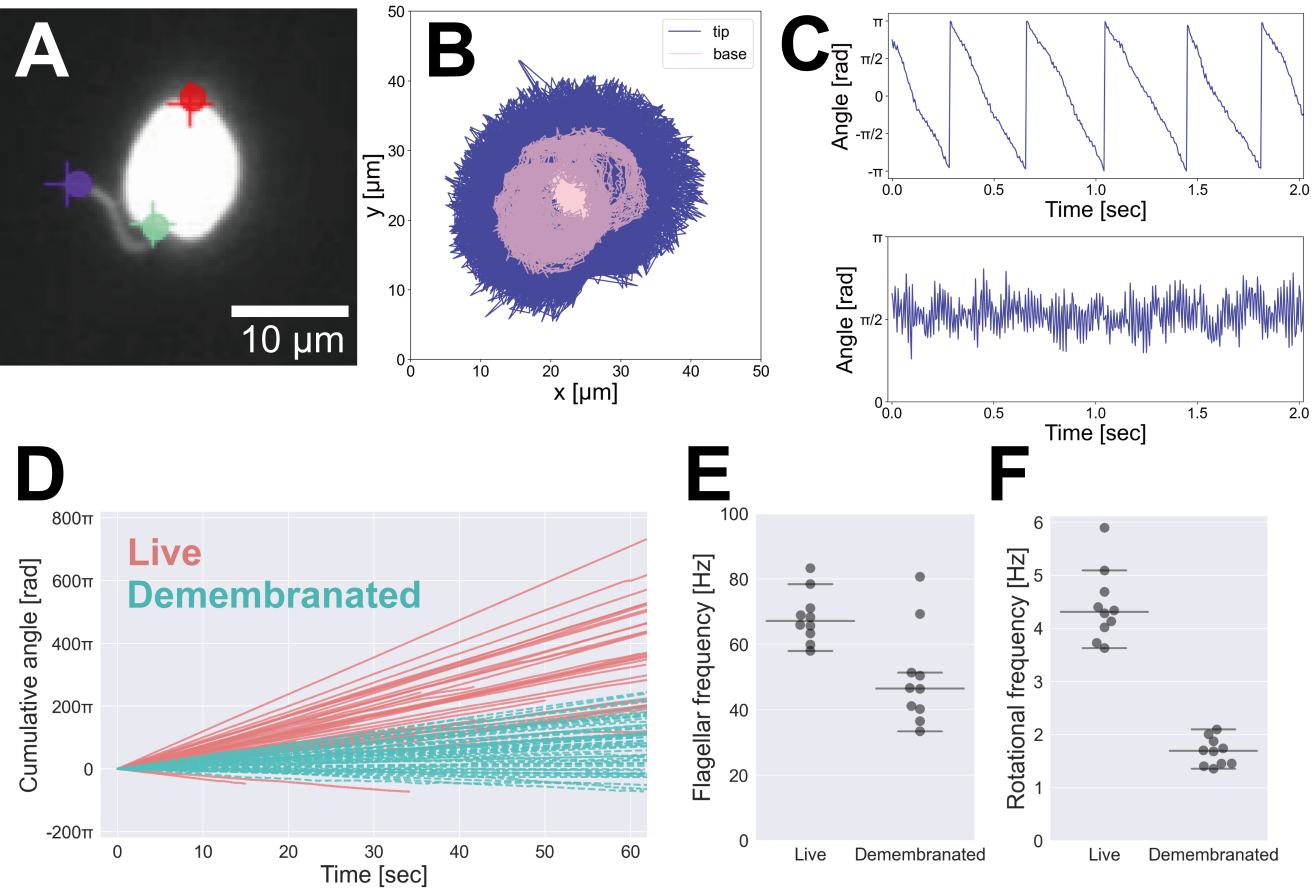
(A) Tracking of body parts using DeepLabCut. Plus marks represent human annotations; circles represent DeepLabCut predictions. Purple: flagellar tip; green: flagellar base; red: cell posterior. (B) Plot showing the positions of the flagellar tip and base over 12,607 frames. Navy: flagellar tip; pink: flagellar base. (C) Time courses of angles: upper panel, body angles; lower panel, flagellar angles. (D) Rotation of live cells (red, solid line) and demembranated models (cyan, broken line). The graph is adjusted so that counterclockwise rotation corresponds to the positive direction. (E) Flagellar beat frequencies of live cells and demembranated models. The horizontal lines represent the median (center line), as well as the minimum (bottom line) and maximum (top line) values that are within 1.5 times the interquartile range from the first quartile (Q1) and the third quartile (Q3). (F) Rotational frequencies of live cells and demembranated models. The horizontal lines are the same as those in E.

## Description

Flagella and cilia are slender structures that extend from eukaryotic cells. Motile flagella and cilia play critical roles in various biological processes, such as enabling swimming in protists (Kage et al. 2024), sperm (Shiba et al. 2008), and planktonic larvae of invertebrates (Yaguchi et al. 2022), as well as generating fluid flows in multicellular organisms (Brooks and Wallingford 2014). Quantifying flagellar motility is essential for understanding the mechanisms underlying these processes.

Previously, flagellar motility was quantified using a semi-automated method called Bohboh (Shiba, Mogami, and Baba 2002), which required manual clicking to define the starting and ending points of flagella. While effective for small datasets, this approach was unsuitable for handling large-scale data due to its labor-intensive nature. To address this limitation, we automated the analysis of flagellar motility using DeepLabCut, a convolutional neural network (CNN)-based tracking tool originally developed for animal behavior studies (Mathis et al. 2018). However, as DeepLabCut is designed to detect discrete points rather than thin structures, it could not directly track the entire length of the flagella. Instead, we successfully tracked the flagellar tip and base as representative points ( Fig. 1A ).

To validate our method, we used the *uni1* mutant of *Chlamydomonas reinhardtii* (Huang et al. 1982) as a model. Unlike wild-type *Chlamydomonas,* which swims using two flagella in a breaststroke-like motion, *uni1* cells possess a single flagellum and exhibit rotational motion with little translational movement. We trained DeepLabCut to annotate three key points on the cell: the flagellar tip, the flagellar base, and the posterior end of the cell ( Fig. 1A ). With this pipeline, we characterized rotational dynamics of live cells and demembranated cell models (Goodenough 1983; Wakabayashi and Kamiya 2015), which are “zombies” that are dead with addition of surfactants and reactivated with addition of ATP.

Figure 1B illustrates the trajectories of the flagellar tip and base of a live cell over 12,607 frames (~1 minute), demonstrating the successful tracking of rotational motion. This plot confirms that the rotational behavior of *uni1* cells was accurately detected.

Figure 1C shows the time courses of body angles (upper panel) and flagellar angles (lower panel) of a live cell. The body angles indicate continuous rotation, while the flagellar angles exhibit periodic oscillations, characteristic of flagellar beating. Using these angle data, we calculated rotational velocities.

Figure 1D presents the cumulative angle trajectories of live cells and demembranated cell models. Among 46 live cells, 44 exhibited counterclockwise (CCW) rotation, while only two (4.3%) showed clockwise (CW) rotation. In contrast, demembranated models displayed a more mixed behavior, with 10 out of 56 cells (17.9%) rotating CW. These results suggest that the directionality of rotation may be influenced by the diameter of the flagella (axoneme) and flagellar waveform. In addition, the rotation of the demembranated models tended to be slower compared to the live cells.

To further characterize the motility, we analyzed the flagellar beat frequency and rotational frequency of the body for the top 10 CCW-rotating cells in both live and demembranated conditions. Using Fourier spectrum peak detection ( Fig. 1E, F), we found that the flagellar beat frequency decreased by approximately 30% in demembranated models compared to live cells (medians: 67.1 Hz in live cells and 46.5 Hz in demembranated models). Rotational frequency of the body, however, decreased by approximately 60% (medians: 4.3 Hz in live cells and 1.7 Hz in demembranated models). This disproportionate reduction suggests that changes in the flagellar waveform, rather than frequency alone, contribute to the altered motility in demembranated models.

While our current method successfully quantifies key aspects of flagellar motility, further improvements are necessary. For instance, integrating deep learning-based techniques to detect both the flagellar tip and base, along with developing image analysis methods capable of tracking the entire flagellum, could substantially enhance the accuracy and efficiency of flagellar and ciliary motility analysis. Although existing algorithms have been developed to detect primary cilia (Lauring et al. 2019) and to automate the analysis of motile cilia (Walker, Ishimoto, and Wheeler 2019; Gallagher et al. 2019), these studies are limited by imaging methods or the number of detectable flagella/cilia. A deep learning-based approach could address these challenges. Such advancements would significantly reduce manual effort and enable more comprehensive investigations into the mechanics of flagella and cilia.

## Methods

**Culture**

*Chlamydomonas reinhardtii* *uni1-1* (CC2506) was obtained from the *Chlamydomonas* Resource Center at the University of Minnesota. The strain was maintained on sterile TAP (tris-acetate-phosphate) agar plates (2%) at 20°C under a 14:10 light/dark cycle. For liquid culture, cells were inoculated into 100 mL of sterile TAP medium and gently aerated at 25°C under a 12:12 light/dark cycle for two days prior to the experiment. All experiments were conducted during the light phase.

**Demembranation**

Demembranation was performed largely following the method described by Wakabayashi and Kamiya (2015). A two-day liquid culture was harvested into disposable 50 mL tubes and centrifuged at 400 g for 3 min using a MX-100 centrifuge (TOMY, Tokyo, Japan). A 1 mL aliquot of the culture was set aside without centrifugation to serve as live cell controls. After centrifugation, the supernatant was discarded, and the pellet was resuspended in 5 mL of HES buffer containing 10 mM HEPES-Na (pH 7.4), 1 mM EGTA and 4% (w/v) sucrose with 1 mM dithiothreitol (DTT). The suspension was centrifuged again at 400 g for 3 min, and the supernatant was discarded. Approximately 500 µL of demembranation solution containing 30 mM HEPES-Na (pH 7.4), 1 mM EGTA, 5 mM MgSO _4_ , 50 mM sodium acetate, 1 mM DTT and 0.1% IGEPAL® CA-630 (9002-93-1, Sigma-Aldrich, St. Louis, MO, USA) was then added to the pellet, and the mixture was gently tapped to ensure thorough mixing. The sample was kept on ice.

A 20 µL aliquot of the treated cells was inspected on a glass slide using an optical microscope (BX50, Olympus, Tokyo, Japan; 10x objective). At this stage, the experimenter confirmed that all cells were non-motile, indicating successful demembranation (referred to as "demembranated models"). Following this confirmation, a reactivation solution containing 30 mM HEPES-Na (pH 7.4), 1 mM EGTA, 5 mM MgSO _4_ , 50 mM sodium acetate, 1 mM DTT, 0.5% (w/v) polyethylene glycol (PEG) 20,000, and 1 mM ATP (51963-61-2, Sigma-Aldrich, St. Louis, MO, USA, pH 7.2) was mixed with the demembranated models at a ratio of 9:1 on ice. The mixture was inspected again under the microscope to verify successful reactivation. Once reactivation was confirmed, the ratio of demembranated models to reactivation solution was adjusted to achieve a final cell density of approximately 1 × 10 ^6^ cells/mL. The amount of demembranated models comprised no more than 1/10 of the total volume. The sample was once again kept on ice.

**Imaging**

The flow chamber was constructed using two 90-µm spacers (double-sided tape NW-5, Nichiban, Tokyo, Japan), placed between a glass slide (S1126, Matsunami, Osaka, Japan) and a coverslip (No. 1, 18 × 18 mm, Matsunami, Osaka, Japan). The sample was introduced into the chamber through the gap between the spacers, and both openings were sealed with vaseline. To prevent flagellar adhesion, the chamber was pre-treated with 100 mg/mL PLL(20)-g[3.5]- PEG(5) (SuSos, Dübendorf, Switzerland) by flowing the solution into the chamber, incubating for 5 minutes, and washing with TAP for live cells or reactivation solution for demembranated models.

The chamber was observed using an upright microscope (BX53, Olympus, Tokyo, Japan; 20x objective, NA 0.5) under dark-field illumination (dry dark-field condenser, U-DCD; halogen lamp, Olympus, Tokyo, Japan). Videos were captured using a high-speed camera (VCC-H1540, Digimo, Tokyo, Japan) at 200 fps for approximately 60 seconds. Observations were conducted at a controlled temperature of ~24°C and completed within 30 minutes after mixing demembranated models and reactivation solution. Since *uni1* cells were adsorbed to the glass surface, recordings were made of cells located beneath the coverslip.

**Image pretreatment**

To prevent misidentification caused by switching between multiple individuals in the multi-animal mode of DeepLabCut, videos were manually reviewed, and spatial regions containing only a single individual were cropped using Fiji ( https://imagej.net/software/fiji/ ). These cropped regions were subsequently used for DeepLabCut training and analysis. To minimize interactions between cells, only images of cells positioned at least 2–3 body lengths apart from others were included in the analysis.

**Model training and image analysis using DeepLabCut**

DeepLabCut (version 2.2.3) was used in single-animal mode. Approximately 100–200 frames from 5 videos were used to train separate models for live cells and demembranated models. Separate training (50,000 iterations for each) was performed because demembranated flagellar axonemes are thinner than intact flagella. Three body parts were annotated: the flagellar tip, the flagellar base, and the posterior end of the cell ( Fig. 1A ). The mean deviations between human annotations and DeepLabCut predictions for test data were 4.59 px for live cells and 2.59 px for demembranated models, where 1 µm = 4.14 px.

Pretreated videos were subsequently analyzed using DeepLabCut. We analyzed 46 videos (representing 46 individuals) from four experiments for live cells and 56 videos (representing 56 individuals) from three experiments for demembranated models.

**Analysis of coordinate data**

Time-series coordinate data were obtained from DeepLabCut analysis. The body angle was calculated based on the coordinates of the flagellar base and the posterior end of the cell. The flagellar angle was defined as the angle between the base-tip vector and the posterior-base vector.

For both live cells and demembranated models, cells exhibiting counterclockwise (CCW) rotation with the top 10 rotational velocities were selected for analysis of rotational frequency and flagellar beat frequency. The direction of rotation and rotational velocities were initially determined from the slope of a least-squares fit to the cumulative angle time course ( Fig. 1D ). In this analysis, the coordinates with a tip likelihood below 0.95 were excluded. The direction of rotation was then verified through visual inspection of the videos. For three movies of demembranated cell models where inconsistencies between the slope analysis and visual inspection were found, the results from visual inspection were adopted. In all such cases, CCW rotation had been misidentified as CW by the slope analysis.

Rotational and flagellar beat frequencies were calculated as follows: time-series angle data were analyzed using fast Fourier transform (FFT), and the peak frequency of the Fourier spectrum was manually identified from peaks detected using the *signal.find_peaks* function in the scipy package in Python.

All movies used in this study are available on Zenodo ( https://doi.org/10.5281/zenodo.15098911 ), and the analysis code is accessible on GitHub ( https://github.com/kageazusa/uni ).

## Data Availability

Description: DeepLabCut tracking of a live uni1 cell. Purple: flagellar tip; green: flagellar base; red: cell posterior. The video is displayed at real-time speed.. Resource Type: Audiovisual. DOI: https://doi.org/10.22002/evty6-wjv18
